# An Effective Simulation Analysis of Transient Electromagnetic Multiple Faults

**DOI:** 10.3390/s20071976

**Published:** 2020-04-01

**Authors:** Liang Dong, Hongxin Zhang, Shaofei Sun, Lei Zhu, Xiaotong Cui, Bablu K. Ghosh

**Affiliations:** 1 School of Electronic Engineering, Beijing University of Posts and Telecommunications, Beijing 100876, China; dongliang@163.com (L.D.); sfsun@bupt.edu.cn (S.S.); cuixiaotong@bupt.edu.cn (X.C.); 2 Communication and Electronic Engineering Institute, Qiqihar University, Qiqihar 161006, China; zhuzhubutterfly@163.com; 3 Faculty of Engineering, University Malaysia Sabah, Kota Kinabalu 88400, Malaysia; ghoshbab@ums.edu.my

**Keywords:** transient electromagnetic injection, ciphertext-only fault analysis, Midori, random multiple fault attacks, differential attack

## Abstract

Embedded encryption devices and smart sensors are vulnerable to physical attacks. Due to the continuous shrinking of chip size, laser injection, particle radiation and electromagnetic transient injection are possible methods that introduce transient multiple faults. In the fault analysis stage, the adversary is unclear about the actual number of faults injected. Typically, the single-nibble fault analysis encounters difficulties. Therefore, in this paper, we propose novel ciphertext-only impossible differentials that can analyze the number of random faults to six nibbles. We use the impossible differentials to exclude the secret key that definitely does not exist, and then gradually obtain the unique secret key through inverse difference equations. Using software simulation, we conducted 32,000 random multiple fault attacks on Midori. The experiments were carried out to verify the theoretical model of multiple fault attacks. We obtain the relationship between fault injection and information content. To reduce the number of fault attacks, we further optimized the fault attack method. The secret key can be obtained at least 11 times. The proposed ciphertext-only impossible differential analysis provides an effective method for random multiple faults analysis, which would be helpful for improving the security of block ciphers.

## 1. Introduction

**Figure 1:**
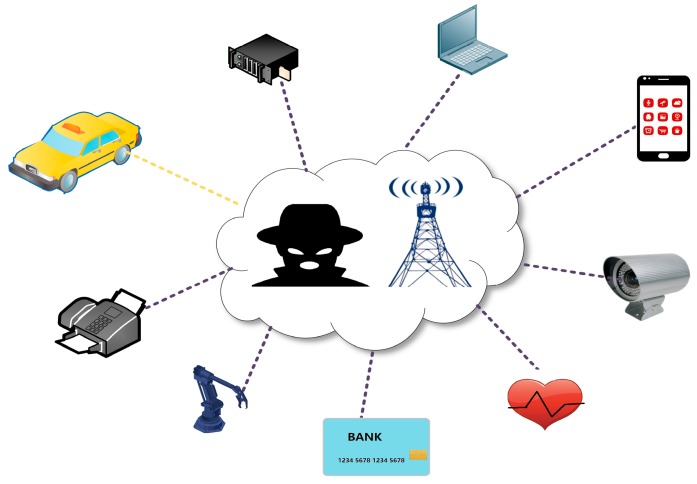
Attack scenario in the Internet of Things (IoT).

With the rapid growth of Internet of Things (IoT) applications, people’s productivity and daily lives have changed. People are enjoying the convenience of intelligent sensor network services; simultaneously, information security is essential. The potential attacks in IoT networks are increasing. The most exposed and vulnerable devices in IoT are routers, cameras, network attached storage (NAS) and printers, as shown in Figure 1. The private data collected by the intelligent sensor networks are carried by the underlying chips and transmitted to networks for data exchanges and data analysis. However, the attacker can obtain the secret key from the chip by performing physical attacks on the target chip. After the fault attacks are injected into the chip, the attackers can steal users’ private data; maliciously attack the network terminal nodes; and monitor and tamper with the sensitive data in the network. Therefore, physical attacks cause considerable harm to smart sensors and embedded encryption devices in the IoT.

Physical attacks have attracted widespread attention in lightweight block ciphers. One method to analyze lightweight block ciphers is directly through electromagnetic radiation using methods such as simple electromagnetic analysis (SEMA) [1], correlation electromagnetic analysis (CEMA) [2] and differential electromagnetic analysis (DEMA) [3]. Other methods involve using clock disturbance, electromagnetic fault injection and laser fault injection [4,5,6] on the specified data registers by analyzing the fault ciphertext to obtain the secret key. Laser fault injection can inject bit-level faults in the specified data register, but the instrument for fault injection is more expensive. However, the manufacturing cost of the electromagnetic fault injection probe is lower. Dehbaoui et al. [7] and S. Ordas et al. [8] designed electromagnetic probes and used electromagnetic attacks to implement bit set or bit reset of data in the chip. Accurately injecting the fault into the encryption device is a prerequisite for obtaining the secret key. After the fault is injected, a proper fault analysis method is required to further obtain the secret key. Since Boneh et al. [9] used fault attacks to break RSA, effective fault analysis methods have become research hotspot. In lightweight block encryption analysis, fault attacks have been extended to impossible differential fault attack (IDFA) [10], impossible differential attack (IDA) [11,12], algebraic fault attack (AFA) [13], impossible meet-in-the-middle attack (IMMA) [14], differential fault attack (DFA) [15] and blind fault attack [16]. These methods mainly analyze the characteristic relationship between the data of the cryptosystem after the injection fault and obtain the secret key by means of solver and mathematical analysis. 

The secret key can be quickly obtained by an appropriate fault analysis model. The proposed fault attack models mainly include the random single-byte [17], random single-nibble model [15], one-bit model [18] and diagonal model [19]. In addition to the fault analysis models proposed above, several novel fault attack models, such as persistent fault attack (PFA) [20] and rebound attack (RA) [21], were proposed. The proposed fault analysis models include a few random multiple fault attack models. However, transient multiple fault attacks occur during an electromagnetic transient fault injection [22,23]. Therefore, studying the random multiple fault attack model has important practical significance for fault analysis. 

Single bit fault [24] and single nibble fault analysis models are widely used in high precision laser fault attacks. However, the attackers can use less sophisticated electromagnetic fault injection to attack sensors in IoT. An electromagnetic fault attack firstly introduces a transient fault [7,8] to the working chip by an electromagnetic probe. The correct key is obtained by collecting and analyzing the relationship between the fault and the correct data. During actual fault injection, the number and location of faults in the data register are affected by the precision of the injection equipment and the electromagnetic interference during the injection. At this time, the output electromagnetic wave injects multiple faults to the target data register. Therefore, the number and locations of random faults in the data register cannot be predicted by the attacker. If a single nibble or single byte model is still used for analysis, the fault attack analysis fails.

Thus, IoT device security can be achieved with a lightweight cryptosystem. Midori is an energy-efficient lightweight cryptosystem proposed by Banik et al. [25]. Midori has broad application prospects in wireless sensor networks. To assess the security of Midori, many researchers developed various attack techniques on Midori. Cheng et al. [15] presented a cell-oriented fault propagation patterns on Midori. Chen et al. [11] designed 10 rounds of impossible differential paths to attack Midori-128. Shahmirzdi et al. [12] conducted three impossible differential attacks on Midori-64 with 10, 11 and 12 rounds. Nozaki et al. [26] distinguished the correct key from the error key by Hamming distance. Todo et al. [27] found a nonlinear invariant Boolean function G to distinguish the secret key.

The differential fault attack (DFA) [15,17,18,19] is a widely applied cryptanalysis technique. The correct and the faulty value are different at the fault point, and the attacker can obtain the secret key by analyzing differential faults. Impossible differential analysis is a powerful analysis method proposed by Knudsen [28] and Biham et al. [29]. By analyzing fault propagation paths, the elements that are absolutely impossible to exist in the key space are eliminated. As such, the correct key is obtained step by step. Differential fault attacks combine well with other attack methods, and the attack effect is significant. Many scholars applied this method to their issues. Combing differential fault analysis with algebraic attack, Jovanovic et al. [30] and Zhao et al. [13] successfully attacked LED-64 with a single fault injection. Li et al. [10] presented a novel impossible differential fault analysis on LED-64. To the best of our knowledge, a random multiple fault attack model on lightweight Midori against the impossible differential fault attack (IDFA) has not been proposed. We further optimized the proposed scheme of fault attacks during the experiment.

The major contributions of the paper are as follows:

(1) We propose an analysis model on lightweight Midori that can be used to analyze most of the random multiple fault attacks. We increase the number of analysis faults from one to six. The random multiple fault attack model can effectively analyze complex fault attacks.
(2) Through experimental simulation analysis, a linear function relationship between the number of fault attacks and the remaining key information content is obtained.
(3) The ciphertext-only fault attack is the attack method with the least known information. In this paper, the secret key is obtained by combining the impossible attack and the differential fault attack. Using the secret key invariant subspace, the secret key can be obtained by intersection of the subspace.

We summarize the results of the best-known attack on Midori-64 in Table 1. Li et al. [31] used six distinguishers to analyze the security of Midori-64. Among them, the hamming weight (HW) distinguisher provides the most effective fault analysis. However, the method can only analyze a random-nibble fault in the 15th round, and the number of fault injections is high. Cheng et al. [15] injected a random-nibble fault into the data register. By analyzing the differential fault propagation path of Midori, they found four invariant fault differential patterns. By analyzing these patterns to estimate the location where the fault was injected, they obtained the secret key. However, Cheng et al. [15] were only able to recover 80% of the secret key, and they did not discuss multiple fault injections. Our proposed method can be used not only analyze multiple random faults but also requires fewer fault injections. At least 11 fault injections are required to obtain the secret key. At present, the problem of multiple faults in data registers has been ignored by researchers. Analyzing the propagation of multiple fault differentials and using the combination of impossible fault attacks and differential fault attacks to improve the security of the lightweight cryptosystem in the case of multiple faults were the motivations of this study.

The rest of this article is divided into the following sections. In Section 2, we briefly describe Midori. In Section 3, we propose a random multiple fault attack model. In Section 4, we provide a detailed calculation for the model. In Section 5, we describe the experimental results. In the last section, we conclude this paper.

## 2. Specifications of Midori and Symbol Description

### 2.1. Midori

**Figure 2:**
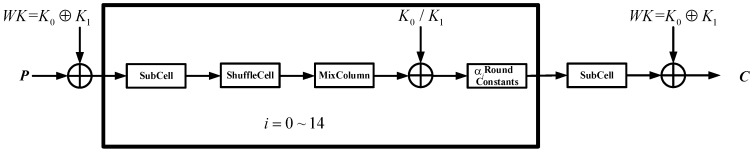
Overall structure of Midori-64.

Midori is an energy-optimized, lightweight cryptosystem that can be used in resource-constrained circuits. Midori-64 and Midori-128 are two cryptosystems with 16 and 20 rounds, respectively. The round function of Midori is KeyAdd, SubCell (SB), ShuffleCell (SC), MixColumn (MC) and Round Constants (RC) in sequence, as shown in Figure 2.

(1) The KeyAdd operation uses the XOR operator with the key. The key of the first round and the last round is the key whitening operation. From the 2nd to the 15th round, $K_{0}$ and $K_{1}$ alternately XORed with the round function in the cryptosystem.
(2) SubCell transform is the only non-linear operation. SubCell operation minimizes the bit flip between input and output. Forward and inverse S-boxes are the same mathematical form.
(3) ShuffleCell rearranges the cell position in a fixed order.
(4) The MixColumn and inverse MixColumn operations are multiplied by the following matrix:
  $$\left(\begin{array}{cc} \begin{array}{cc} 0 & 1\\ 1 & 0 \end{array} & \begin{array}{cc} 1 & 1\\ 1 & 1 \end{array}\\ \begin{array}{cc} 1 & 1\\ 1 & 1 \end{array} & \begin{array}{cc} 0 & 1\\ 1 & 0 \end{array} \end{array}\right)$$
(5) Round Constants operation is XORed by the form of 4 × 4 binary matrices.

### 2.2. Symbol Description

The following notation is used to describe the analysis of Midori. Let C be the right ciphertext and $C^{*}$ be the faulty ciphertext. Let $X^{L}\in {\left({\left\{0,1\right\}}^{4}\right)}^{16}$, $Y^{L}\in {\left({\left\{0,1\right\}}^{4}\right)}^{16}$, $Z^{L}\in {\left({\left\{0,1\right\}}^{4}\right)}^{16}$ and $W^{L}\in {\left({\left\{0,1\right\}}^{4}\right)}^{16}$ denote the output value of the Round Constants, SubCell, ShuffleCell and MixColumn layers in the L-th round with 1≤L≤ 16, respectively. Let $\Delta X^{L}$, $\Delta Y^{L}$, $\Delta Z^{L}$ and $\Delta W^{L}$ denote the output difference of *X*, *Y*, *Z* and *W* in the L-th round, respectively. Equation (1) denotes each nibble in $\Delta X^{L}$, $\Delta Y^{L}$, $\Delta Z^{L}$ and $\;\Delta W^{L}$, respectively.

(1) $$\left\{ \begin{array}{c} \Delta X^{L}=(\Delta x_{\left\{1,1\right\}}^{L},\Delta x_{\left\{i,j\right\}}^{L},\cdots ,\Delta x_{\left\{4,4\right\}}^{L})\Delta Y^{L}=(\Delta y_{\left\{1,1\right\}}^{L},\Delta y_{\left\{i,j\right\}}^{L},\cdots ,\Delta y_{\left\{4,4\right\}}^{L})\Delta Z^{L}=(\Delta z_{\left\{1,1\right\}}^{L},\Delta z_{\left\{i,j\right\}}^{L},\cdots ,\Delta z_{\left\{4,4\right\}}^{L})\Delta W^{L}=(\Delta w_{\left\{1,1\right\}}^{L},\Delta w_{\left\{i,j\right\}}^{L},\cdots ,\Delta w_{\left\{4,4\right\}}^{L}) \end{array}\right.\;i=1\cdots 4\;\;,\;\;j=1\cdots 4.$$

Let {i,j} denote the i-th row and the j-th column. ⊕ denotes bitwise exclusive-or operation. We denote the inverse operations of Round Constants, SubCell, ShuffleCell, MixColumn by  INVRC,INVSB, INVSHC  and INVMC, respectively. Let $\emptyset_{\left\{i,j\right\}}$ and $\emptyset_{\left\{j\right\}}$ denote the set of $Y_{\left\{i,j\right\}}^{16}$ and $Y_{\left\{j\right\}}^{16}$ when the estimated $\Delta Z_{\left\{i,j\right\}}^{15}=0$ and $\Delta Z_{\left\{j\right\}}^{15}$ = 0, respectively. Let θ denote the intersection of ρ.

## 3. Random Multi-Fault Attack Model

**Figure 3:**
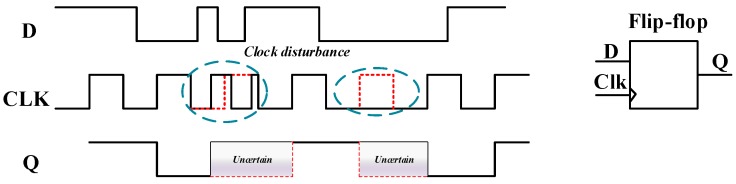
Clock disturbance of D flip-flop.

Space particle radiation, aging of electronics and electromagnetic interference can disturb the current inside a chip. Faults are classified into intentional injection faults and unintentional injection faults. Faults can also be classified as transient faults, permanent faults and persistent faults. Compared with persistent fault injection [20], transient fault injection causes less damage to the chip. Therefore, most of the fault attacks are transient fault attacks. Although the position of the internal bit flip is related to the accuracy of the fault injection tool, the faults mentioned above have more random faults in the actual fault injection and attackers do not know. The electromagnetic waves radiated by the probe can disturb the clock circuit and the surrounding registers. Data transmission and data exchange in the chip are clock synchronized. In data transmission, setup-time and hold-time must be stable. In the process of electromagnetic fault injection, clock stability rapidly decreases, so setup-time and hold-time deviate, as shown in Figure 3. The occurrence of random multiple faults is complicated and ubiquitous. In the process of actual fault attacks analysis, we encounter very complex problems.

A suitable attack model is important for security analysis. If a fault attack model cannot effectively analyze actual faults, the analysis of a cryptosystem will encounter many difficulties. At present, models for multiple fault attacks are lacking. Liao et al. [32] proposed a multiple fault attack model with no more than three bytes. Using matrix diagonals, Saha et al. [19] analyzed multiple byte faults, but with relatively few types of faults. To the best of our knowledge, no random multiple fault attack model against Midori has yet been proposed. From the perspective of engineering, in this paper, a general random multiple fault model is proposed. The random multiple fault analysis model can be applied to most of the random fault attacks and improve the security of lightweight cryptosystems in IoT networks.

### 3.1. Fault Attacks Hypothesis

**Figure 4:**
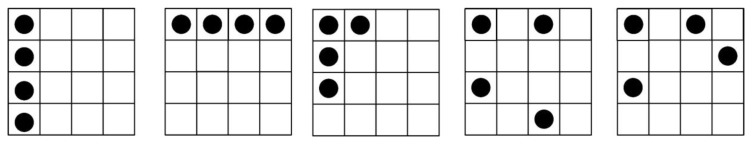
Four random faults.

**Figure 5:**
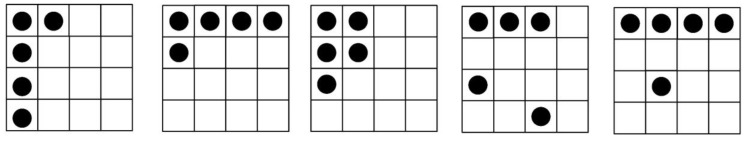
Five random faults.

**Figure 6:**
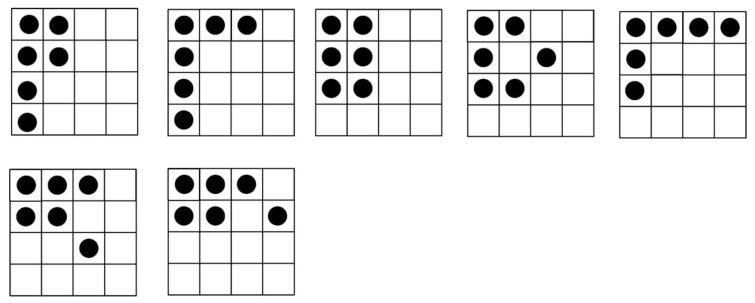
Six random faults.

This paper does not deal with the physical implementation of the attack. Fault attacks against Midori can be implemented based the following assumptions: An attacker is able to inject faults in the 14th round data register of Midori-64 and the number of the faults is no more than six. There is no fault injection in other memory elements of the crypto-hardware. After fault injection, the value of fault registers changes and the fault location is unknown. The attacker can obtain the correct and the faulty ciphertexts after each fault injection. The attacker does not need to know the correct plaintext, so this is a ciphertext-only attack. An attacker injects multiple random faults in the 14th round of cryptosystem. As can be seen from Figure 2, the fault injection is the same in SubCell, ShuffleCell, and Round Constants. Assuming an electromagnetic fault is injected, there are four to six nibble faults. Random faults in data registers are shown in Figure 4, Figure 5 and Figure 6. The black circle indicates the fault point. Figure 4, Figure 5 and Figure 6 describe the distributions of four to six faults. The faults in Figure 4, Figure 5 and Figure 6 indicate that the probability of fault occurrence of each position of the column is the same; there are zero-four faults in each column. 

### 3.2. Analysis of Random Multiple Fault Attack Models

**Figure 7:**
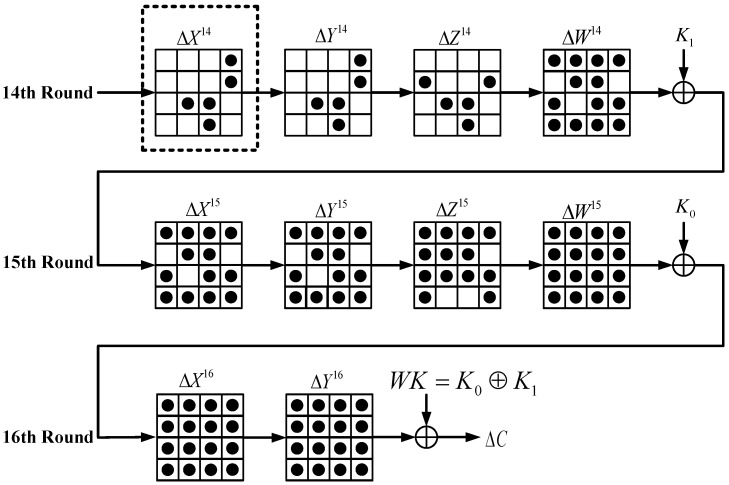
Random multiple faults’ propagation paths.

Figure 7 shows how faults propagate when random multiple faults are injected into the 14th round. The fault state in the dashed box can be replaced by any state in Figure 4, Figure 5 and Figure 6. According to the fault injection assumption mentioned above, the number of random faults in each column of the $\Delta X_{}^{15}$,$\;\Delta Y_{}^{15}\;$ and $\Delta Z_{}^{15}$ is 0 to 4. To successfully implement random multiple fault attacks, we explain the two problems.

1. Problem 1: The location of no faults in each column of $\Delta X_{}^{15}$, $\Delta Y_{}^{15}$ and $\;\Delta Z_{}^{15}$ is unknown. There are four positions in a column. We estimate all four fault positions as fault-free, and then take the union of the estimated invariant space in the column.
2. Problem 2: As the location and number of each fault injection are unknown, there may be 0–4 faults in a certain column of $\Delta Z_{}^{15}$. After injecting random multiple faults two or three times into the cryptosystem, an adversary takes a fault-free union at each position of $\Delta Z_{}^{15}$. It is possible that the number of faults in a column is four. If the adversary predicts this column as fault-free, an error will occur. To avoid mistakes, the adversary can inject faults two or three times into the cryptosystem. In other words, the adversary avoids mistakes by taking the unions multiple times. We provide a detailed explanation using the following differential fault attack equations.

## 4. Analysis of Fault Difference Equations

The analysis of the multiple differential fault path is shown in Figure 7. We perform the inverse operation through the inverse output differential of the 16th round S-box.

(2) $$\Delta Y_{}^{16}=\Delta C_{}^{16}$$

By observing the Figure 7, we obtain the following differential equations:

(3) $$\Delta W_{}^{15}=\Delta X_{}^{16}=\mathrm{INVSB}\left(\Delta Y_{}^{16}\right)$$

(4) $$\Delta Z_{}^{15}=\mathrm{INVMIX}\left(\Delta W_{}^{15}\right)$$

We can further obtain Equations (5)–(8) by expanding Equation (4).

(5) $$\Delta Z_{\left\{1,1+l\right\}}^{15}=\mathrm{INVSB}\left(\Delta C_{\left\{2,1+l\right\}}^{16}\right)⊕\mathrm{INVSB}(\Delta C_{\left\{3,1+l\right\}}^{16})⊕\mathrm{INVSB}(\Delta C_{\left\{4,1+l\right\}}^{16})$$

(6) $$\Delta Z_{\left\{2,1+l\right\}}^{15}=\mathrm{INVSB}\left(\Delta C_{\left\{1,1+l\right\}}^{16}\right)⊕\mathrm{INVSB}(\Delta C_{\left\{3,1+l\right\}}^{16}⊕\mathrm{INVSB}(\Delta C_{\left\{4,1+l\right\}}^{16})$$

(7) $$\Delta Z_{\left\{3,1+l\right\}}^{15}=\mathrm{INVSB}\left(\Delta C_{\left\{1,1+l\right\}}^{16}\right)⊕\mathrm{INVSB}(\Delta C_{\left\{2,1+l\right\}}^{16}⊕\mathrm{INVSB}(\Delta C_{\left\{4,1+l\right\}}^{16})$$

(8) $$\Delta Z_{\left\{4,1+l\right\}}^{15}=\mathrm{INVSB}\left(\Delta C_{\left\{1,1+l\right\}}^{16}\right)⊕\mathrm{INVSB}(\Delta C_{\left\{2,1+l\right\}}^{16})⊕\mathrm{INVSB}(\Delta C_{\left\{3,1+l\right\}}^{16})$$

(9) $$\emptyset_{\left\{j\right\}}=\emptyset_{\left\{1,j\right\}}\cup^{\text{​}}\emptyset_{\left\{2,j\right\}}\cup^{\text{​}}\emptyset_{\left\{3,j\right\}}\cup^{\text{​}}\emptyset_{\left\{4,j\right\}}$$

where *j* = 1,2,3,4 and  l=0,1,2,3.

The relationship between $\Delta Z^{15}$ and $\Delta Y^{15}$ is shown in Table 2.

For the differential characteristics of the inverse S-box, as shown in Table 3, one input difference corresponds to multiple output differences. However, in a fault attack, the plaintext and secret key are always the same; that is, we can uniquely determine $Y_{}^{16}$ and $Y_{}^{15}$ by the estimation of S-box outputs.

The adversary estimates $\Delta W^{15}$ are listed in Table 2. Predicting the locations of multiple faults is impossible due to the complexity of random multiple fault injection. When the differential faults propagate to $\Delta Z_{}^{15}$, the adversary estimates the fault-free position of $\Delta Z_{}^{15}$, using Equations (5)–(9). Invariant space $\;Y_{}^{16}$ is reduced by excluding non-zero nibbles in each column of $\Delta Z_{}^{15}$. According to the explanations of Problems 1 and 2 above, when the adversary injects faults two or three times, the fault-free difference must exist in some columns of $\Delta Z^{15}$. After a fault attack, each column of candidate $Y^{16}$ can be expressed by the Equations (10)–(12). According to Problems 1 and 2 discussed above, the attackers independently induce faults two or three times at the 14th round and take the union of ∅, as shown in Equation (10).

(10) $$\rho_{m}=\emptyset_{3m-2}\cup^{\text{​}}\emptyset_{3m-1}\cup^{\text{​}}\emptyset_{3m}\left(m=1,2,3\right)$$

where 3m−2, 3m−1 and 3m represent the number of fault attacks and m represents the number of ∅ unions. The attacker can obtain the set of estimated $Y^{16}$, as shown in Equations (11) and (12).

(11) $$\theta_{p}=\rho_{2p-1}\cap^{\text{​}}\rho_{2p}\left(p=1,2,\cdots \right)$$

(12) $$\omega_{q}=\theta_{2q-1}\cap^{\text{​}}\theta_{2q}\left(q=1,2,\cdots \right)$$

where p  and q  are the number of intersections. The attacker injects faults repeatedly until the element in $\omega_{q}$ is unique. During fault attacks, the elements of the set θ may be an empty set for various reasons. When θ is empty, the attacker cancels ρ and re-injects random faults. Therefore, the adversary will eventually obtain a unique $Y^{16}$ by constantly injecting faults.

(13) $$\mathrm{SB}\left(Y^{16}\right)⊕K_{0}⊕K_{1}=C$$

Then, WK can be obtained according to the following formula:

(14) $$WK=K_{0}⊕K_{1}=C⊕\mathrm{INVSB}\left(Y^{16}\right).$$

According to the key schedule of Midori, the adversary makes further derivations to obtain $K_{0}$. The equations are shown in Equations (15) to (19).

(15) $$\Delta Y^{15}=\;\mathrm{INVSHC}(\mathrm{INVMIX}\left(\Delta X^{16}\right))$$

(16) $$\Delta X^{16}=\;\mathrm{INVSB}\left(C⊕WK\right)⊕\mathrm{INVSB}\left(C^{*}⊕WK\right)$$

With the method proposed above, we do not need to inject the fault again; the unique $Y^{15}$ can be recovered by the same fault attack data.

(17) $$W^{15}=\;\mathrm{MC}\left(\mathrm{SHC}\left(\mathrm{SB}\left(Y^{15}\right)\right)\right)$$

(18) $$\mathrm{SB}\left(\mathrm{RC}\left(W^{15}⊕K_{0}\right)\right)⊕WK=C$$

$K_{0}$ can be obtained using Equation (19).

(19) $$K_{0}=W^{15}⊕\mathrm{INVRC}(\mathrm{INVSB}(C⊕WK))$$

## 5. Experimental Analysis and Results

The random multiple fault attacks experiments were performed on a PC with a Core^TM^ i3 CPU with 4GB of RAM, using MATLAB language. 

**Figure 8:**
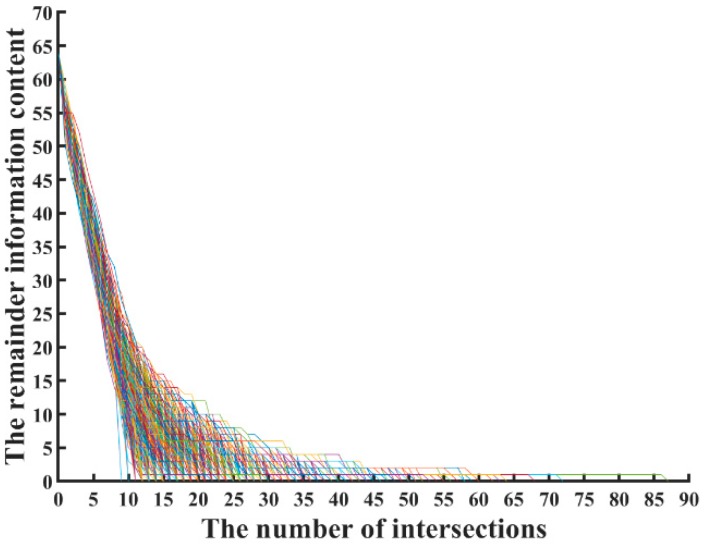
The remaining information content in 32,000 fault attacks.

Information entropy is a method used to measure the estimation of source data. Sakiyama et al. [33] theoretically analyzed information entropy of the key leakage on S-box. However, in the multi-fault analysis for lightweight Midori, reports are absent on the secret key leakage relationship between the number of fault attacks and leak information content. To determine the relationship between the number of fault attacks and information content, we simulated 32,000 random multiple faults. Figure 8 shows a total of 32,000 curves, each colored line representing a fault attack process of the recovery secret key. Midori-64 initially needed to determine the 64-bit secret key without fault injection. With our proposed algorithm, when the intersection of the secret key space was taken about 10 times, the amount of undefined information content was reduced to five. We continued to intersect the secret key space set ρ; the remainder of the information content gradually reduced until all the secret keys were recovered.

**Figure 9:**
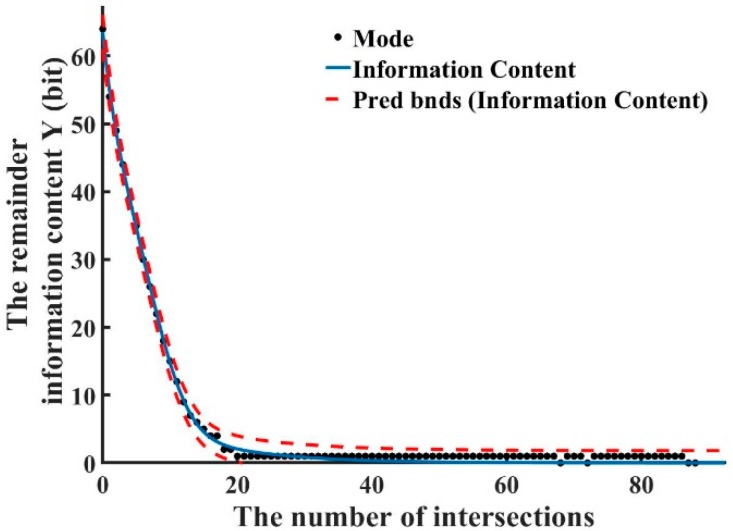
The relationship between the remaining information content and the number of intersections.

To identify the relationship between predicted fault injection and the amount of information to be predicted, we took the mode (black dot) simulation fitting during 32,000 fault attacks and obtained the functional relationship as shown below. Compared with Figure 8, the fitted graph in Figure 9 perfectly depicts the entire fault analysis process. The red curve in Figure 9 shows the boundary values of the data during the prediction process. The relationship between the number of intersections and remaining information content secret key bits is shown in Figure 9. We obtained the following formula by computer fitting:

(20) $$y=\{\begin{array}{cc} a\cdot e^{-{\left(\frac{n-b}{c}\right)}^{2}} & 0\leq n\leq 26\\ \left[0,3\right] & n\geq 27 \end{array},$$

where y is the remaining information content; n is the number of intersections; and a, b and  c are constants. The ranges of a, b  and c are: a=105.4 (78.25, 132.6),b=−10.85 (−14.56,−7.142) and c=14.9 (13.18,16.63).

Therefore,

(21) $$y=\{\begin{array}{cc} \mathrm{105.4}\cdot e^{-{\left(\frac{n+\mathrm{10.58}}{\mathrm{14.9}}\right)}^{2}} & 0\leq n\leq 26\\ \left[0,3\right] & n\geq 27 \end{array}$$

**Figure 10:**
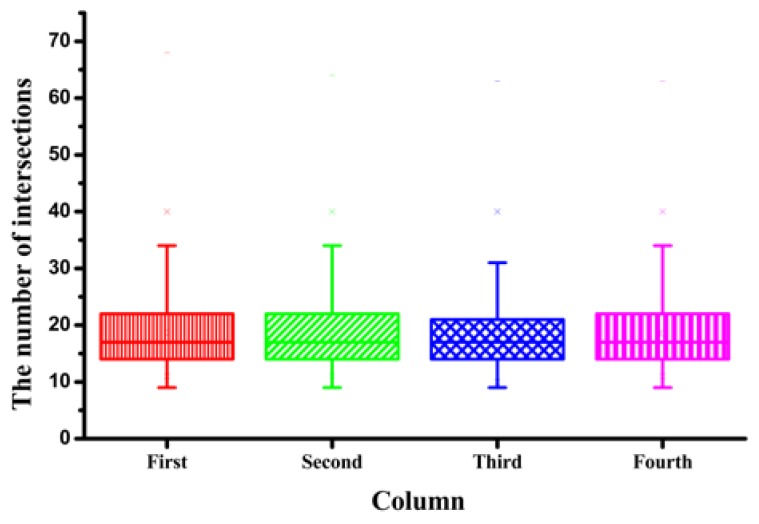
The number of attacks of each column.

Equation (20) shows good agreement with the experimental results. The adversary can obtain the remaining information content by taking the number of intersections into Equation (20). The remaining information content shrinks with the intersection, as shown in Figure 9. When the remaining information content is 1 bit, more faults need to be injected to make the information content 0 bit. To further explain the existence of a large number of 1-bits, we counted the remaining information content of each column during the attacks. As shown in Figure 10, the number of intersections is around 20.

To reduce the number of fault injections and improve attack efficiency, the attacker stops fault injection when there is 1 bit left. We took the undetermined secret key into Midori-64 for verification.

**Figure 11:**
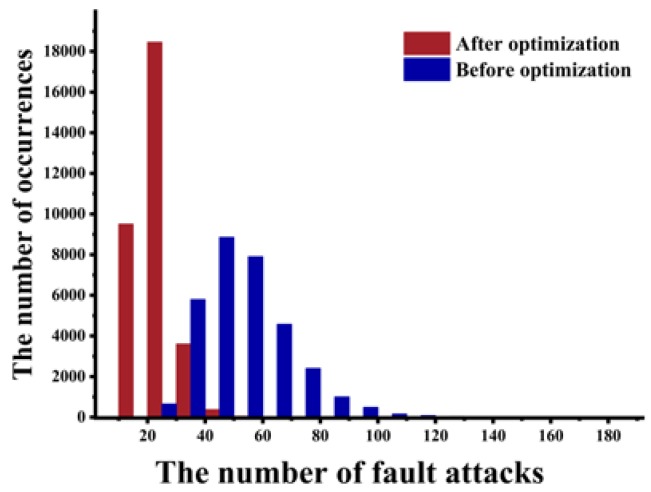
The relationship between the remaining information content and the number of intersections.

**Figure 12:**
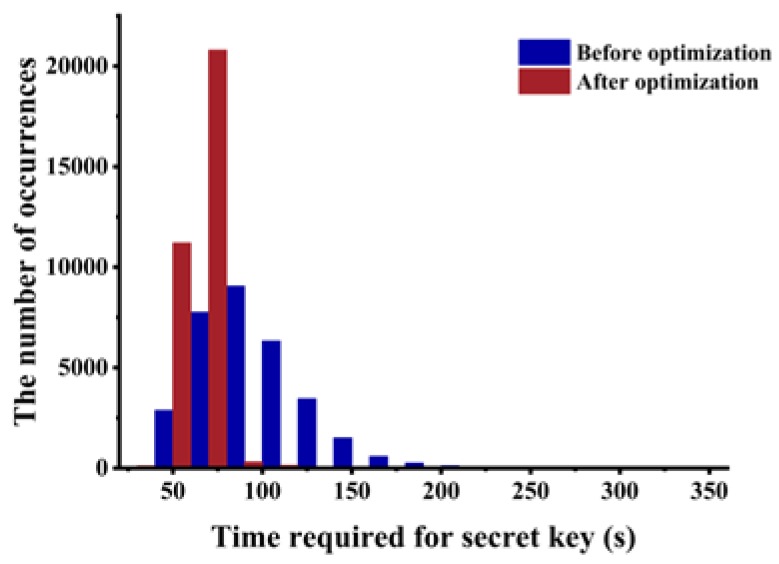
The distribution of the attack time.

As shown in Figure 11, we counted the number of fault attacks before and after optimization. After optimization, the number of fault attacks reduced considerably, and most of attacks could be implemented within 20 times. Figure 12 shows the time distribution of fault attack. Most of the security keys can be recovered within 80 s. The efficiency after optimization greatly improved compared to before optimization.

## 6. Conclusions

In IoT networks, the data security of each sensor node faces severe challenges. The scattered distribution of a large number of nodes is convenient for attackers. In this paper, a novel random multiple fault attack method on Midori is proposed. The fault attack method can successfully recover the secret key in Midori with at least 11 attacks. Using computer simulation, we obtain the leakage relationship between the number of fault attacks and information content, which provides a theoretical basis for quickly obtaining the secret key. An adversary can use this function to judge the range of the remaining secret key. The random multiple fault attack method, provided in this paper is applicable to many fields. We present the random multiple fault analysis method, which provides a theoretical model for the analysis of unknown location and the number of fault injections. The proposed attack model can be applied to most of the laser fault attacks and electromagnetic fault attacks. We further optimized the attack scheme, reduced the number of fault attacks and decreased the time of fault attacks. The method proposed in this paper is helpful for analyzing the gradual process for obtaining secret keys under multiple faults. We expect that the multiple fault attacks will improve the security of lightweight cryptosystems.

## Figures and Tables

**Table 1:** Comparison of this work with previous fault attacks on Midori-64.

Reference	Fault Model					Number of Faults
	Method	Probability	Round	Value	Distinguish multiple- nibble fault	
[31]	HW	100%	R = 15	1 Nibble	No	280
[15]	DFA	80%	R = 14	1 Nibble	No	2
this paper	IDFA	100%	R = 14	1–6 Nibble(s)	Yes	11

**Table 2:** The inverse differential of Midori.

$\Delta Y_{0}^{15}$	$\Delta Y_{1}^{15}$	$\Delta Y_{2}^{15}$	$\Delta Y_{3}^{15}$	$\Delta Y_{4}^{15}$	$\Delta Y_{5}^{15}$	$\Delta Y_{6}^{15}$	$\Delta Y_{7}^{15}$	$\Delta Y_{8}^{15}$	$\Delta Y_{9}^{15}$	$\Delta Y_{10}^{15}$	$\Delta Y_{11}^{15}$	$\Delta Y_{12}^{15}$	$\Delta Y_{13}^{15}$	$\Delta Y_{14}^{15}$	$\Delta Y_{15}^{15}$
$\Delta Z_{0}^{15}$	$\Delta Z_{7}^{15}$	$\Delta Z_{14}^{15}$	$\Delta Z_{9}^{15}$	$\Delta Z_{5}^{15}$	$\Delta Z_{2}^{15}$	$\Delta Z_{11}^{15}$	$\Delta Z_{12}^{15}$	$\Delta Z_{15}^{15}$	$\Delta Z_{8}^{15}$	$\Delta Z_{1}^{15}$	$\Delta Z_{6}^{15}$	$\Delta Z_{10}^{15}$	$\Delta Z_{13}^{15}$	$\Delta Z_{4}^{15}$	$\Delta Z_{3}^{15}$

**Table 3:** Difference distribution of Midori inverse S-box.

γ	β
1	1 2 4 5 6 8 14
2	1 4 9 12
3	4 6 7 8 9 13 15
4	1 2 3 4 5 8 11
5	1 4 7 9 10 12
6	1 3 7 8 12 13 15
7	3 5 6 11 13 14
8	1 3 4 6 9 11 12 14
9	2 3 5 8 9 11 12
10	5 10 13 15
11	4 7 8 9 11 13 15
12	5 6 8 9 12 14
13	3 6 7 10 11 14
14	1 7 8 12 13 14 15
15	3 6 10 11 14 15
0	0

γ and β represent inverse input and output difference, respectively.
